# Case Report: Can Targeted Intraoperative Radiotherapy in Patients With Breast Cancer and Pacemakers be the New Standard of Care?

**DOI:** 10.3389/fonc.2022.927174

**Published:** 2022-07-12

**Authors:** Fardeen Bhimani, Kelly Johnson, N. Patrik Brodin, Wolfgang A. Tomé, Jana Fox, Keyur Mehta, Maureen McEvoy, Sheldon Feldman

**Affiliations:** ^1^ Breast Surgery Division, Department of Surgery, Montefiore Medical Center, Montefiore Einstein Center for Cancer Care, Bronx, NY, United States; ^2^ Department of Radiation Oncology, Montefiore Medical Center, Bronx, NY, United States

**Keywords:** breast cancer, intraoperative radiotherapy, pacemaker, case report, TARGIT, breast, invasive duct breast cancer (IDC)

## Abstract

**Background:**

Partial breast irradiation with Intra-operative radiotherapy (IORT) has become a popular management option as opposed to whole breast radiation using external beam radiotherapy for breast cancer patients. While previous studies have highlighted the use of IORT in breast cancer patients, there is a scarcity of literature on the use of IORT in those who also have ipsilateral pacemakers. Thus, the aim of our case report is to highlight the applicability of IORT in breast cancer patients who also have a pacemaker.

**Case Reports:**

Two female patients with an implanted dual-chamber pacemaker presented with a diagnosis of left-sided invasive ductal carcinoma on mammogram. Mammography of the left breast revealed a 10 mm and 7 mm spiculated mass, respectively, further confirmed with an ultrasound-guided core biopsy that was conclusive of clinical Stage I T1 N0 grade 2, ER +, PR + Her2 – invasive ductal carcinoma. They met our eligibility criteria for IORT, which is being performed as a registry trial. These patients underwent a wide excision lumpectomy along with IORT.

**Conclusion:**

Our findings underscore the successful use of targeted IORT for breast-conserving surgery in a patient with invasive ductal carcinoma and pacemaker, hence eliminating the necessity for relocating pacemaker surgeries in these patients. Furthermore, no device failure or malfunction for the pacemaker was recorded before, during, or after the surgery, demonstrating the safety of using IORT in patients with preinstalled pacemaker despite a lack of evidence on safe radiation dosage or manufacturer guidelines. Nonetheless, the effects of IORT on pacemaker < 10 cm were not studied in our patients and further clinical studies are recommended to reinforce the applicability and safe distance of IORT in breast cancer patients with pacemaker.

## Introduction

Breast cancer is the most common newly diagnosed malignancy among women across the United States (1). Of all the cancers diagnosed in women, breast cancer accounts for about 1/3^rd^ of the cases (2). The incidence of breast cancer increases roughly by 0.5% per year, and approximately 290,560 patients will be diagnosed with breast cancer in 2022 (1, 2). Nonetheless, many treatment options for breast cancer management have arisen over the years. While the majority of patients who opt for breast conservation receive whole-breast irradiation (WBI), there has been a change in preference toward partial breast irradiation (PBI) delivered over 5 days. Additionally, intra-operative radiotherapy (IORT) allows for a single dose to be administered at the time of lumpectomy. Furthermore, because of the intrinsic benefit of tissue preservation and breast conservation, IORT has become a popular choice for patients (3–6). Prior study by the TARGIT group has demonstrated that the short-range kilovoltage energy sources minimize the radiation dosage to normal tissues by eliminating the electromagnetic radiation and scattered radiation typical of external beam radiotherapy (EBRT) while also being safe and effective (7). A parallel randomized trial on 2298 patients by the same group found that TARGIT-IORT was non-inferior to EBRT, with a local recurrence rate of 2.11% for TARGIT-IORT vs. 0.95% for EBRT (8). Furthermore, using the same patients, the same group reported long-term outcomes evaluating the impact of tumor size, grade, receptor status, and lymph node status on local recurrence-free survival, as well as the impact of local recurrence on distant relapse and mortality (9). They found no difference in 5-year local recurrence-free survival between TARGIT-IORT and EBRT in any tumor subgroup. Additionally, they reported that TARGIT-IORT significantly reduced non-breast cancer mortality, even when patients received supplemental EBRT, with a Hazard Ratio (HR) of 0.38 (95% CI 0.17–0.88, p = 0.0091) (9).

A caveat to the use of IORT is presumed to be patients with preinstalled cardiac pacemakers. This may relate to the fact that the ionizing radiation interferes with the modern cardiac pacemaker that has complementary metal oxide semiconductor circuitry (CMOS). The number of cardiac pacemakers implanted worldwide has increased dramatically, from approximately half a million in 2002 to nearly a million in 2016 (10–12). In the United States, approximately 200,000 pacemakers are implanted in bradycardic patients each year (13). In an older population, the increasing overlap between these two groups is anticipated to contribute to an increase in the number of instances involving people with pacemaker and breast cancer (12). There is a paucity of literature looking at the use of IORT in patients with pacemakers, and the manufacturers are less likely to conduct cardiac pacemaker testing with various IORT devices due to a large number of devices available on the market for both (14). Thus, our case reports aim to highlight the applicability of IORT in breast cancer patients who also have a pacemaker on the same side.

## Case Reports

### Patient 1

**Figure 1:**
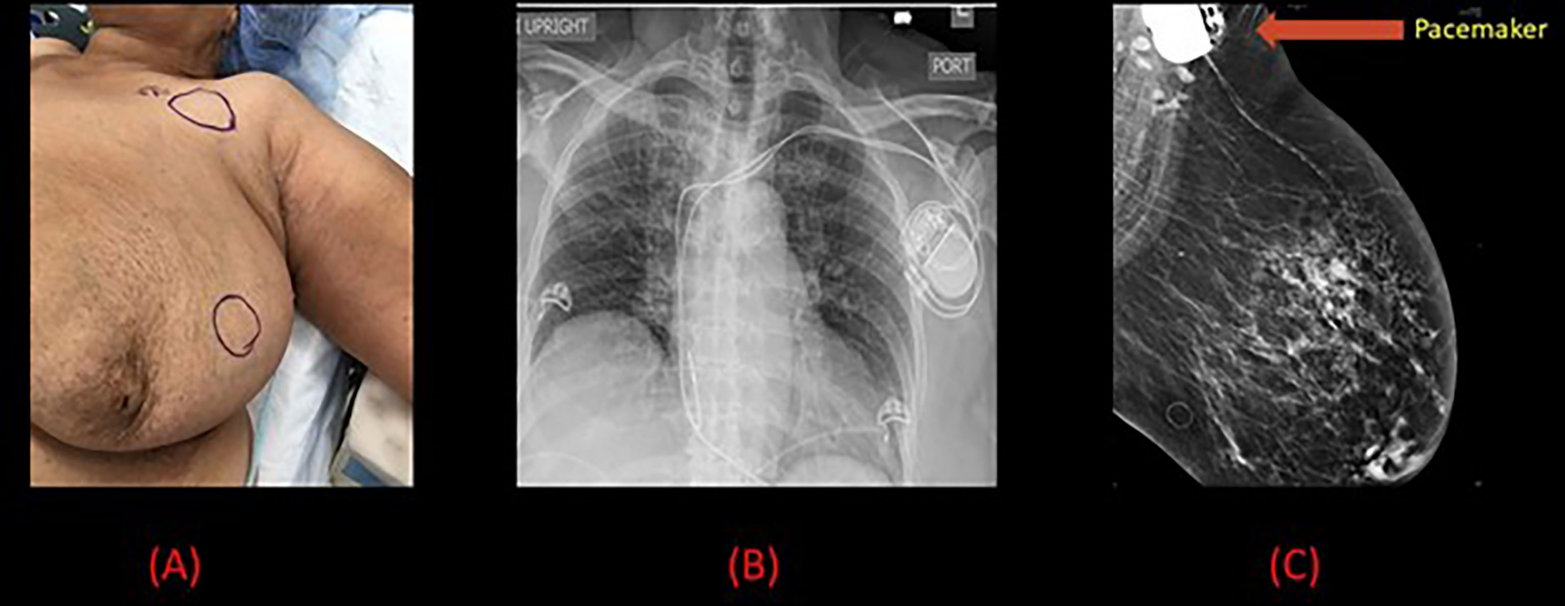
Pre-operative and radiological images for patient 1. **(A)** Pre-operative image of the left breast with the location of the pacemaker marked by a circle in the superior portion close to the neck and the inferior circle indicates the presence of radiological seed. **(B)** Chest x-ray demonstrating left sided pacemaker with dual leads projecting over the right atrium and right ventricle. **(C)** Mammogram demonstrating a 10 mm spiculated mass in the left breast along with the pacemaker.

An 82-year-old female presented with a lump in her left breast for 1-month post-dual-chamber pacemaker implantation for sinus bradycardia. Mammography of the left breast revealed a 10 mm spiculated mass noted in the middle depth of the left breast at approximately the 2:00 axis (**Figure 1**). Subsequently, an ultrasound guided core biopsy revealed clinical Stage I T1 (10 mm) N0 grade 2, ER +, PR + Her2 – invasive ductal carcinoma. She had a PMHx of chronic kidney disease, diabetes mellitus, hypercholesterolemia, hypertension, hypothyroidism, venous insufficiency, and a dual-lead pacemaker (DDD mode, model Biotronik type Edora DR-T ProMRI) implanted for sinus bradycardia. The pacemaker was programed to a lower rate of 60 bpm and a maximum of 130 bpm. The pacemaker was placed anatomically in a subcutaneous pocket in the upper pole of the left breast, 10 cm away from the primary tumor.

During the multidisciplinary meeting, the patient’s condition was discussed. Due to the proximity of the tumor to the pacemaker, and keeping the patient’s comorbidities in mind, it was collectively decided that a localized left breast lumpectomy along with targeted IORT will be offered.

The IORT would be delivered by the intrabeam system using targeted intraoperative radiotherapy (TARGIT) technique which is administering IORT through a spherical applicator placed in the tumor bed for a time duration determined by the applicator’s diameter (15). As a result, IORT avoids the requirement for the pacemaker to be relocated, which would have been necessary prior to external beam radiotherapy. Prior to the surgery, she underwent placement of an iodine-125 seed by Radiology under mammographic guidance to localize the tumor and sentinel lymph node biopsy was not performed since it would not affect her management.

**Figure 2:**
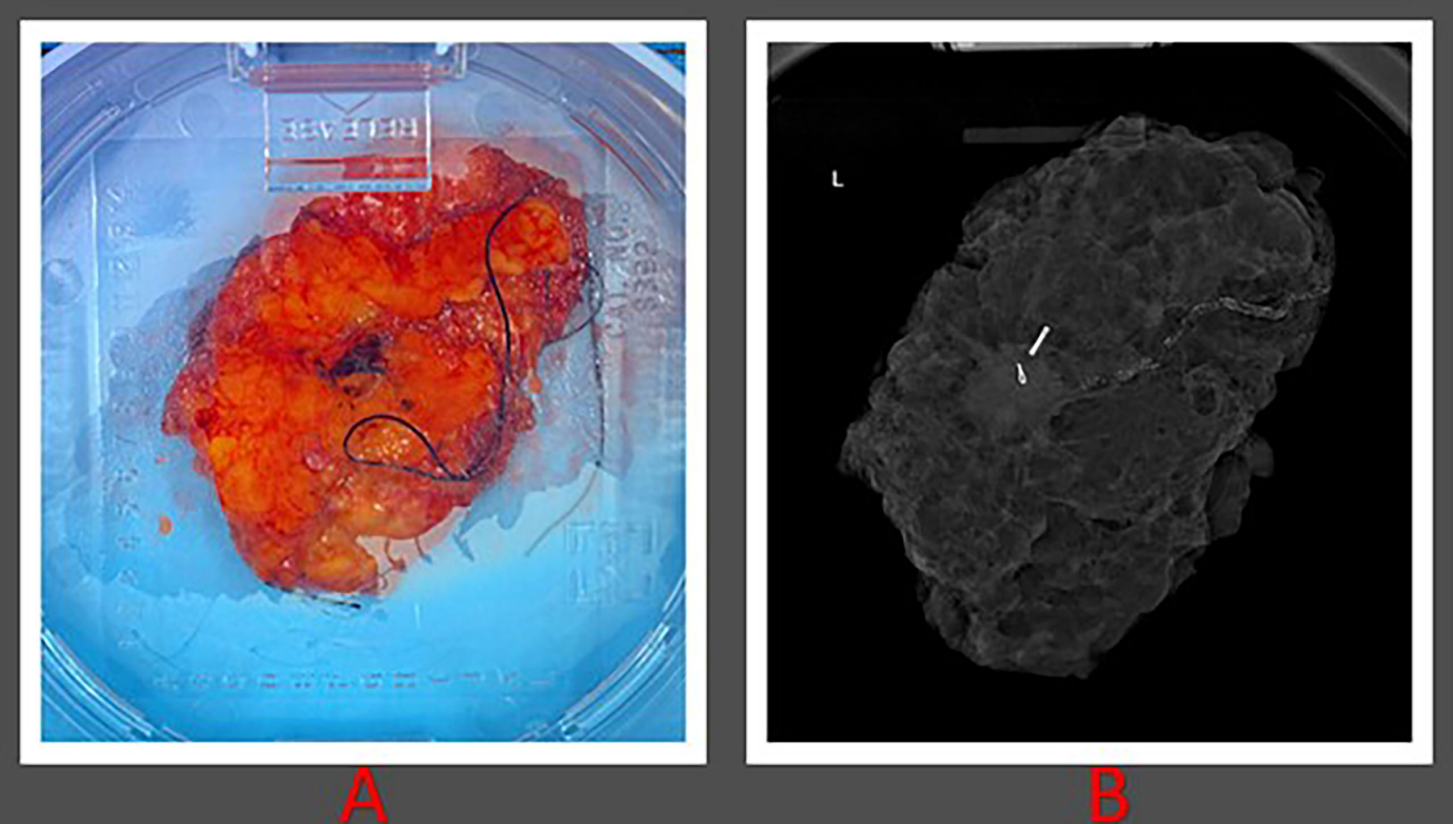
Histopathological images of patient 1. **(A)** Gross lumpectomy specimen measuring 10 cm in dimension extending from superior to inferior margin and measuring 6.2 cm extending from lateral to medial margin. **(B)** Lumpectomy specimen with presence of radiological seed.

Wide local excision was performed and TARGIT-IORT was delivered during surgery using the Intrabeam 600 (Zeiss, Oberkochen, Germany) (**Figure 2**). Ultrasound was then used to measure the distance from the applicator to the skin with the closest bridge being inferior at 10.6 mm. IORT was delivered with a 40-mm diameter spherical applicator, delivering 20 Gy to the surface of the surgical margin in direct contact with the applicator for a duration of 25 minutes. *In vivo* dosimetry was performed by placing an optically stimulated luminescence dosimeter (OSLD) under the radiation shield on the skin surface in close proximity to the pacemaker site during treatment. The measured absorbed dose resulting from the Zeiss INTRABEAM IORT system radiation on the skin surface in close proximity to the pacemaker site was measured to be 0.23 Gy, using the same methodology described previously by our group (16).

The surgery and TARGIT procedure were both successful. The patient tolerated the procedure very well, and there was no malfunction of the pacemaker device throughout the surgery or IORT. The patient made an uneventful recovery and was sent home the same day. Histology confirmed the presence of an 18 mm grade 2 invasive ductal carcinoma with admixed lobular features that had been fully removed with clear margins. The tumor tested positive for ER/PR but negative for Her-2. As part of her adjuvant endocrine therapy, a treatment plan with aromatase inhibitor was decided.

### Patient 2

**Figure 3:**
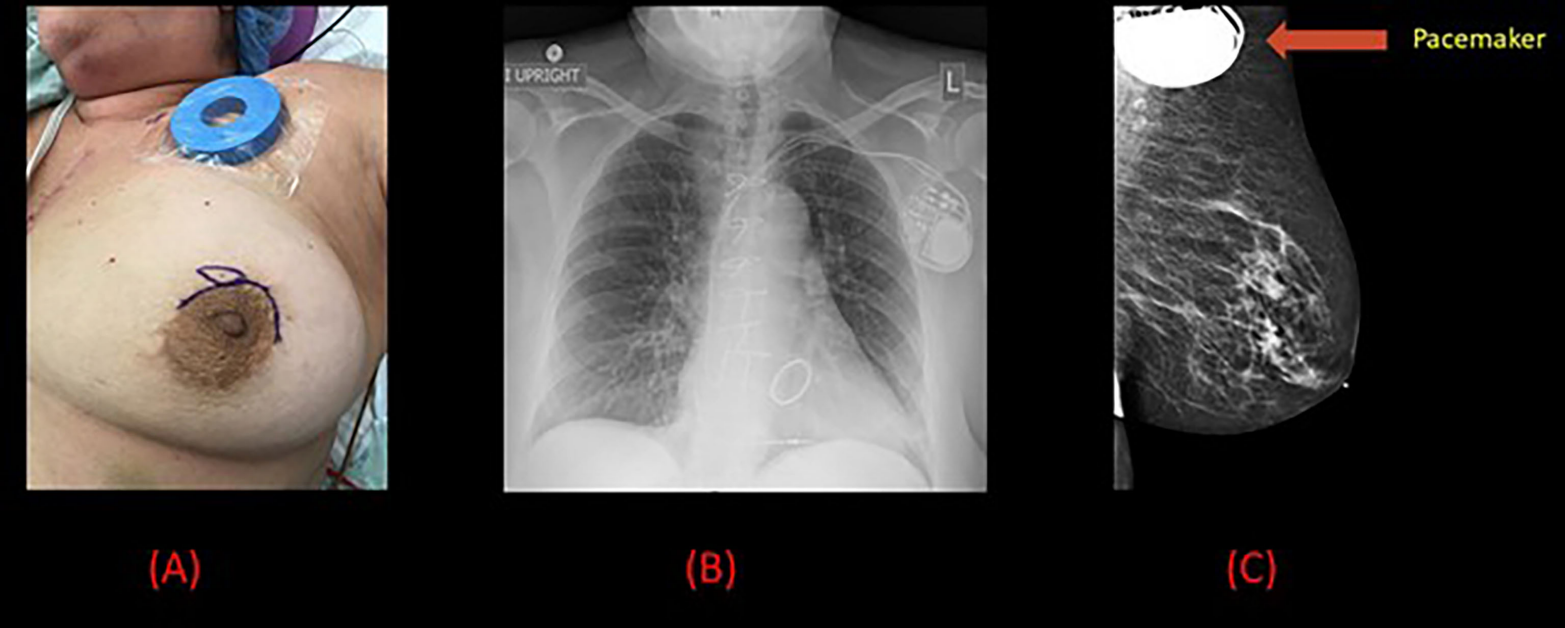
Pre-operative and radiological images for patient 2. **(A)** Pre-operative image of the left breast with a magnet placed on the pacemaker and surgical marking indicating the tumor site. **(B)** Chest x-ray demonstrating left sided pacemaker with leads projecting over the right atrium and right ventricle. **(C)** Mammogram demonstrating a 7 mm spiculated mass in the left breast along with the pacemaker.

A 64-year-old female with a history of surgically managed right invasive lobular carcinoma with lumpectomy, sentinel lymph node biopsy, and whole breast radiation 6 years back presented with an incidental finding of a 7 mm spiculated mass in the anterior to middle depth at 12:00 axis of the left breast on a routine follow-up mammogram (**Figure 3**). An ultrasound guided core biopsy revealed clinical stage I T1 (7 mm) N0 grade 2, ER +, PR + Her2 – invasive ductal carcinoma and a focal ductal carcinoma *in situ* (DCIS), nuclear grade 2, cribriform type. The patient also had a history of atrial fibrillation, hepatitis C, asthma, chronic obstructive pulmonary disease (COPD), mitral stenosis, pulmonary hypertension, mitral valve replacement, cerebrovascular accident, and dual-lead pacemaker (DDD mode, model Medtronic type AZURE XT DR MRI) implanted for Mobitz type II heart block. The pacemaker was programed to a lower rate of 60 bpm and a maximum of 130 bpm. Anatomically, the pacemaker was placed in a subcutaneous pocket in the upper pole of the left breast, 10 cm away from the primary tumor.

Patient underwent wide local excision followed by TARGIT-IORT during surgery delivered with the help of an Intrabeam 600. The distance from the applicator to the skin was then measured using ultrasound, with the closest bridge being inferior at 20 mm. IORT was delivered with using a 35-mm diameter spherical applicator, which delivered 20 Gy to the surface of the surgical margin in direct contact with the applicator for a duration of 16 minutes. During treatment, an OSLD was placed on the skin surface beneath the radiation shield, close to the pacemaker site, for *in vivo* dosimetry. The measured absorbed dose from the Intrabeam IORT system radiation on the skin surface near the pacemaker site was estimated to be 0.185 Gy.

Both the surgery and the TARGIT procedures were successful. The procedures were well tolerated by the patient, and there was no malfunction of the pacemaker device during surgery or IORT. The patient made an uneventful recovery and was discharged the same day. Histology confirmed the presence of a 12 mm grade 2 invasive micropapillary carcinoma excised with clear margins. The tumor tested positive for ER/PR but negative for Her-2.

## Discussion

The effect of radiation therapy on implanted electronic devices such as pacemaker is difficult to estimate (17, 18). The dose contribution from scatter during radiotherapy with high-energy megavoltage beams, are the primary concern for pacemakers if they are not directly in the radiation treatment field. Radiation often affects the device’s random access memory (RAM), which can have a detrimental influence on device function and, in higher amounts, may permanently damage electrical components (19). However, this is rarely seen unless the device is in close proximity to radiation. Radiation-induced electromagnetic interference may shut the magnetic reed switch, resulting in an unanticipated reversion to an OFF mode, necessitating restoration to therapy mode (19). Because of these concerns, EBRT utilization is not recommended in the proximity of the pacemaker, with many surgeons choosing to remove the complete system and reimplant on the opposite side to clear the area for surgery and eventual radiation therapy, despite the risk of lead extraction (20–23). Contrastingly, few prefer to reimplant the pacemaker from the prepectoral pocket into the neck pocket *via* small cervicotomy, which allows the device to be far away from the radiation site (24). Many of these concerns are bridged by the use of IORT. Since most pacemaker are placed on the left side, the findings of our study underscore the utility of IORT, particularly in the management of the left sided breast cancer.

Another limitation for the use of EBRT is increased non-breast cancer related mortality rate when compared to TARGIT-IORT (8). WBI has proven to have an increased incidence of second cancers and heart diseases (25–27). The incidence of lung cancer mortality without breast cancer recurrence ≥ 10 years after radiotherapy had a rate ratio of 2.10 (95% confidence interval (CI), 1.48 to 2.98; P = <0.001) on the basis of 134 cancer patients (25). Furthermore, EBRT has been proven to cause various heart diseases including ischemic heart disease, coronary stenosis, myocardial infarction, valvular disease, pericarditis and other cardiac abnormalities (25–27). However, these complications are negated using IORT which significantly decreases the non-breast cancer related mortality rate (45 vs. 74 events for TARGIT-IORT and EBRT, respectively, hazard ratio 0.59; 95% CI, 0.40 to 0.86; P=0.005), including the cardiovascular causes (8). As a result, IORT may be a prudent choice of treatment, particularly in patients with pre-existing cardiac conditions such as pacemaker.

IORT allows a single high dose of radiation to be delivered directly to the surgical margins shortly after tumor excision, and the very low energy (50 kVp) minimizes scatter and radiation exposure to the surrounding tissues, which in turn reduces the risk of electromagnetic interference (4, 28, 29). IORT can lead to higher patient compliance owing to shorter treatment duration and fewer visits, which may overall lead to better patient experience (30). Additionally, patients with locally recurrent breast cancer who have previously undergone breast conserving surgery with EBRT can undergo a second breast-conserving surgery with IORT as an alternative to mastectomy (31). Furthermore, a previous randomized control trial conducted by the TARGIT group demonstrated that TARGIT-IORT is both safe and efficacious (7). Contrastingly, there is no current consensus on the use of IORT in patients with breast cancer and concomitant pacemaker, and surgeons remain cautious because pacemaker failures due to radiation can theoretically occur at any dose (32). As a result, many of them opt to use surgery and adjuvant therapy to treat these patients (33). Furthermore, elderly patients, such as patient 1, who was 82 years old, could benefit from surgery and endocrine therapy while avoiding radiotherapy (34). The decision to give this patient radiotherapy was based on the Cancer and Leukemia Group B (CALGB) 9343 trial which found that adding radiation therapy to endocrine therapy improved locoregional recurrence in women age ≥ 70 years (35). Kunkler et al. (36) also randomized 1,326 patients with non-metastatic hormone receptor-positive breast cancer who were at least 65 years old, had breast-conserving surgery, and were receiving adjuvant hormone therapy. The authors reported that the rate of local recurrence after 10 years was significantly higher in patients who did not receive radiation therapy compared to patients who did (9.8% vs. 0.9%), supporting our decision to treat this patient with radiotherapy.

Although previous studies have evaluated the utility of IORT in patients with breast cancer, fewer have elucidated its use when associated with a presence of ipsilateral pacemaker (7, 14, 33). Chen et al. (29) assessed the dose for a patient with a pacemaker being treated in the left breast with IORT in phantom cases. They found that as the radial distance between the applicator and the pacemaker increases, the radiation exposure to the pacemaker decreases. In their study, despite the radiation dose being increased to 10-20 Gy, no pacemaker device malfunctions, or failures were observed. Additionally, Keshtgar et al. (33) reported a case of left-sided invasive ductal carcinoma of the breast with a pacemaker in an elderly woman. They reported a tumor-to-pacemaker distance of 9 cm that was successfully treated with IORT. Over the course of 26 minutes, they delivered a radiation dose of 20 Gy to the surgical margin and 6 Gy to an area 1 cm away from the surgical margin. Similarly, in both of our patients who were successfully treated with IORT, the tumor-to-pacemaker distance was 10 cm. In addition, a 20 Gy radiation dose was delivered to the surgical margin in both patients, and an absorbed dose of 0.23 Gy and 0.185 Gy to the skin at the pacemaker site were measured, respectively. Thus, our findings add to the limited existing literature on use of IORT in breast cancer patients with ipsilateral pacemaker. Furthermore, our findings show that successful radiotherapy can be delivered without the need for the pacemaker to be reimplanted on the contralateral side or in a neck pocket. To support the widespread applicability of IORT as a treatment modality in breast cancer patients with pacemaker, further clinical studies are needed.

The use of such single high-dose radiation delivered directly to the surgical margins in patients with pacemaker is a step in the right direction. However, from a practical standpoint, it is unclear how much radiation exposure to the pacemaker a clinician should consider acceptable. The American Association of Physicists in Medicine (AAPM) Task Group 203 (TG-203) recently recommended that radiation exposure to the pacemaker up to 2 Gy is considered low risk, while exposures greater than 5 Gy are considered high risk (37). Moreover, the threshold for radiation exposure for pacemaker devices varies from each manufacturer. Medtronic, Inc. (Minneapolis, MN, USA), St. Jude Medical (St. Paul, MN, USA), Biotronik SE & Co (Berlin, Germany), and Boston Scientific (Boston, MA, USA) are the primary manufacturers of pacemaker in the United States. The maximum dose threshold for Medtronic varies from 1 Gy to 5 Gy depending on the model of pacemaker (33, 38), 20-30 Gy for St. Jude Medical (39, 40), and not specified for both Biotronik SE & Co (41) and Boston Scientific (42). Due to the large volume of products available in the market, we explicitly recommend future studies determining the optimal and maximum threshold dose safe for each device as well as safe distance from pacemaker to establish new guidelines.

## Conclusion

Our findings underscore the successful use of targeted IORT for breast-conserving surgery in a patient with invasive ductal carcinoma and pacemaker, hence eliminating the necessity for reimplant pacemaker surgeries in these patients. Furthermore, no device failure or malfunction for the pacemaker was recorded before, during, or after the surgery, demonstrating the safety of using IORT in patients with preinstalled pacemaker despite a lack of evidence on safe radiation dosage or manufacturer guidelines. Nonetheless, the effects of IORT on pacemaker < 10 cm were not studied in our patients and further clinical studies are recommended to reinforce the applicability and safe distance of IORT in breast cancer patients with pacemaker.

## Data Availability Statement

The original contributions presented in the study are included in the article/supplementary material. Further inquiries can be directed to the corresponding author.

## Ethics Statement

Ethical review and approval were not required for the study on human participants in accordance with the local legislation and institutional requirements. The patients/participants provided their written informed consent to participate in this study. Written informed consent was obtained from the individual(s) for the publication of any potentially identifiable images or data included in this article.

## Author Contributions

FB: substantial contributions to acquisition of data and involved in drafting and revision of the manuscript. KJ and MM: substantial contributions in revising the manuscript. NB, WT, JF, and KM: equal contributions to acquisition of data and in the revision of the manuscript. SF: substantial contributions to data acquisition, involved in drafting and revising the manuscript. All authors contributed to the article and approved the submitted version.

## Conflict of Interest

The authors declare that the research was conducted in the absence of any commercial or financial relationships that could be construed as a potential conflict of interest.

## Publisher’s Note

All claims expressed in this article are solely those of the authors and do not necessarily represent those of their affiliated organizations, or those of the publisher, the editors and the reviewers. Any product that may be evaluated in this article, or claim that may be made by its manufacturer, is not guaranteed or endorsed by the publisher.
